# Oral manifestations, salivary flow rates and Candida species in Thai HIV-infected patients 

**DOI:** 10.4317/jced.55384

**Published:** 2019-02-01

**Authors:** Aroonwan Lam-ubol, Sorasun Rungsiyanont, Piamkamon Vacharotayangul, Kraisorn Sappayatosok, Oitip Chankanka

**Affiliations:** 1 Faculty of Dentistry, Srinakharinwirot University, 114 Sukhumvit 23, Wattana, Klongtoey Nua, Bangkok 10110, Thailand; 2 Faculty of Dentistry, Rangsit University, 52/347 Phaholyothin Road, Mueang Pathum Thani District, Pathum Thani 12000, Thailand; 3 Faculty of Dentistry, Prince of Songkla University, 15 Karnjanavanich Raod, Hat Yai, Songkhla 90110, Thailand

## Abstract

**Background:**

Effects of various Highly Active Antiretroviral Therapy (HAART) regimens on oral heath are unclear. Objectives: We aimed to evaluate effects of HAART on oral manifestations, salivary flow rates (SFR) and Candida species in HIV-infected patients who took mostly non-protease inhibitor-based HAART regimens.

**Material and Methods:**

A cross-sectional study was performed on HIV-infected patients taking and never taken HAART who attended Thai Red Cross AIDS Research Centre (n = 48). Non-HIV subjects were recruited as control (n = 20). Oral conditions and salivary flow rates were evaluated using oral examination and measurement of unstimulated and stimulated saliva. In addition, Candida colonization counts (colony forming units; CFUs) and Candida species from the collected saliva were evaluated using CHROMagar.

**Results:**

The most common oral manifestation in HIV-infected subjects taking HAART was hyperpigmentation. Unstimulated and stimulated SFR among the three groups were not statistically significant. Candida colonization was detected in 64%, 65% and 35% of HIV-infected subjects taking HAART, HAART-naïve, and non-HIV subjects, respectively. While 20% and 35% of HIV-infected subjects with and without HAART, respectively, had Candida CFUs higher than 500/ml, all non-HIV carriers had Candida CFUs lower than 500/ml. The most common Candida colonization species was C. albicans in HAART and non-HIV groups. Interestingly, HAART-naïve group was colonized more by non-albicans species.

**Conclusions:**

HAART has minimal effects on oral health. While HAART may not prevent Candida colonization, it might lead to reduction of non-albicans species. Because maintaining low Candida counts is important, HAART administration and antifungal sensitivity test should be considered in HIV-infected patients.

** Key words:**HIV, Candida, HAART, Oral manifestation, Salivary flow rates.

## Introduction

Human Immunodeficiency Virus (HIV) infection has been one of the major public health problems. According to UNAIDS, it was estimated that 36.9 million people were living with HIV worldwide in 2017. Although HIV infection caused high mortality and morbidity in the past, the disease has been better controlled since the introduction of Highly Active Antiretroviral Therapy (HAART) in 2000 (1-3). HIV-infected patients are now living longer. Trends of diseases have been changed with significant decrease in opportunistic infections. However, patients living with AIDS face other health problems such as lipodystrophy, cardiovascular diseases and cancers, due to the disease itself and side effects from HAART (4). Oral side effects of HAART have been reported, such as hyperpigmentation, salivary gland hypofunction, salivary gland enlargement and human papillomavirus infection (1,5). Since then, there have been changes in HAART regimen to maximize antiviral effects and minimize adverse effects. Non-nucleoside reverse transcriptase inhibitor (NNRTI)-containing regimens offer superior virological suppression and better immunological outcome than protease-inhibitor (PI)-containing regimens (2,6,7). Most current first line regimens worldwide, including Thailand, had shifted from PI-containing regimens to NNRTI-containing regimens (1,2,8,9).

Oral candidiasis, one of the most common opportunistic infection in HIV-infected patients, has been significantly reduced with HAART (1-3). Effects of HAART on *Candida* colonization in HIV-infected patients is still controversial (8-10). Although *Candida* species can normally colonize in the oral cavity without clinical symptoms, increased numbers of *Candida* colonization had been shown to promote risk of oral candidiasis (11,12). Oral cavity colonization by *Candida* species can be found in healthy population, however, percentage of *Candida* carriers in HIV-infected patients were reported to be higher than in healthy population (8). *Candida albicans* is the most common species found in oral candidiasis and *Candida* colonization in HIV-infected subjects. However, there has been an increase in non-albicans species in this population (13-15). PIs were shown to have inhibitory effects against *Candida* (16). However, NRTIs and NNRTIs are used more frequently nowadays. Previous studies showed inconsistent results about incidences in developing oral candidiasis between NNRTI-users and PI-users (2,17). In addition, effects of HAART on *Candida* colonization and *Candida* species were unclear.

## Objectives

Studies on effects of HAART on oral changes, *Candida* species and salivary gland function had been reported from several countries, including Thailand, with various results (1,18). These could be because differences in study population, HAART regimens and study methods. The objective of this study was to evaluate effects of HAART on oral manifestations, *Candida* colonization species and salivary flow rates in Thai HIV-infected patients.

## Material and Methods

-Study design and participants

This was a cross-sectional study performed in patients infected with HIV who attended the Thai Red Cross AIDS Research Centre. Both patients receiving HAART and never received HAART (HAART-naïve) were recruited. In addition, control group included non-HIV-infected subjects recruited from the Faculty of Dentistry, Srinakharinwirot University, Bangkok, Thailand. The study protocol was approved by the ethical review boards of the Faculty of Medicine, Chulalongkorn University and Faculty of Dentistry, Srinakharinwirot University. All subjects were informed of the objectives and study protocol, and gave written consent prior to participate in the study. All non-HIV-infected subjects received blood test to confirm HIV-seronegativity. Subjects who had oral candidiasis or systemic or local treatment with antifungals within the previous three months were excluded from the study.

-Data collection

The following data were collected from the HIV-infected subject’s medical records: personal information, medical history and results from laboratory tests (the closest ones to the time of sample collection), CD4 counts, viral load, diagnosis of AIDS, antiretroviral therapy, and use of antifungal or other medication. Subjects were instructed not to eat or drink for one hour prior to sample collection. Subjects were then interviewed regarding dental problems. Oral and dental structures were examined and recorded by oral medicine specialists. Un-stimulated and mechanical-stimulated saliva samples were collected by using standard protocol. For un-stimulated saliva, the subjects were asked to rinse their mouth with water. Then, after swallowing all the saliva present in the mouth, they were instructed to allow new saliva to accumulate in the mouth, and expectorate it into a 50 ml tube that was previously weighed every 60 seconds for a period of 5 minutes. For stimulated saliva, subjects were asked to chew on a piece of paraffin (1 gram) and expectorate whole stimulated saliva into a 50 ml tube for 5 minutes. Salivary flow rates were calculated as ml/min. After collection, the saliva was kept at 4 degrees Celsius and used for analysis within 6 hours.

-Analysis of *Candida* counts and species

The saliva samples were centrifuged and supernatants were used for the analysis. Each saliva sample was diluted 1:10 with phosphate buffered saline pH 7.2 and cultured on CHROMagar *Candida* plates (CHROMagar Microbiology) in an incubator at 37 degrees Celsius. Colony forming units (CFUs) and colony color and morphology were evaluated after 48 hours. CFUs were classified as low (less than 500 CFUs/ml) and high (more than 500 CFUs/ml). *Candida* colonies were characterized based on color according to the manufacturer’s recommendation (*C. albicans*: green, *C. tropicalis*: metallic blue, *C. krusei*: pink, fuzzy, other species: white to mauve). Plates exhibiting no growth were incubated for another 24 hours to confirm the absence of *Candida* colonies.

-Statistical analysis

Demographic data and salivary flow rates of HIV-infected subjects taking HAART, HIV-infected subjected not taking HAART (HAART-naïve) and non-HIV-infected subjects were compared using Independent Sample Kruskal-Wallis test, Mann-Whitney U test or Chi-square as specified.

## Results

-Demographic data of the study population

A total of sixty-eight subjects were included in the study. Twenty-five (36.8%) and 23 (33.8%) of them were HIV-infected patients who received HAART and HAART-naïve, respectively. Twenty (29.4%) non-HIV-infected subjects were included as control group.

Average age and sex of subjects in the three groups were not statistically significant ( Table 1). Duration of HIV infection ranged from 2 to 18 years with an average of 8.5 years in a group taking HAART and 6.7 years in a HAART-naïve group. Average CD4 counts in a HIV-infected groups taking HAART and HAART-naïve were 513+193 and 425+183 cells per ml, respectively. Although CD4 counts in HAART-naïve group were significantly lower (*p*=0.03), all subjects had CD4 counts more than 200 cells per ml. As expected, average viral loads in HIV-naïve subjects (16,285 copies per ml) were higher than HIV-infected subjects taking HAART (lower than 40 copies per ml). Regarding HAART regimens, majority (88%) of HIV-infected subjects received combination of NRTIs and NNRTIs. Only 3 HIV-infected subjects (12%) received combination of NRTIs and PIs. Average duration of HAART usage was 5.7 years, ranging from 0.6-13.8 years. Most subjects (80%) had taken HAART for longer than 5 years.

**Table 1:**
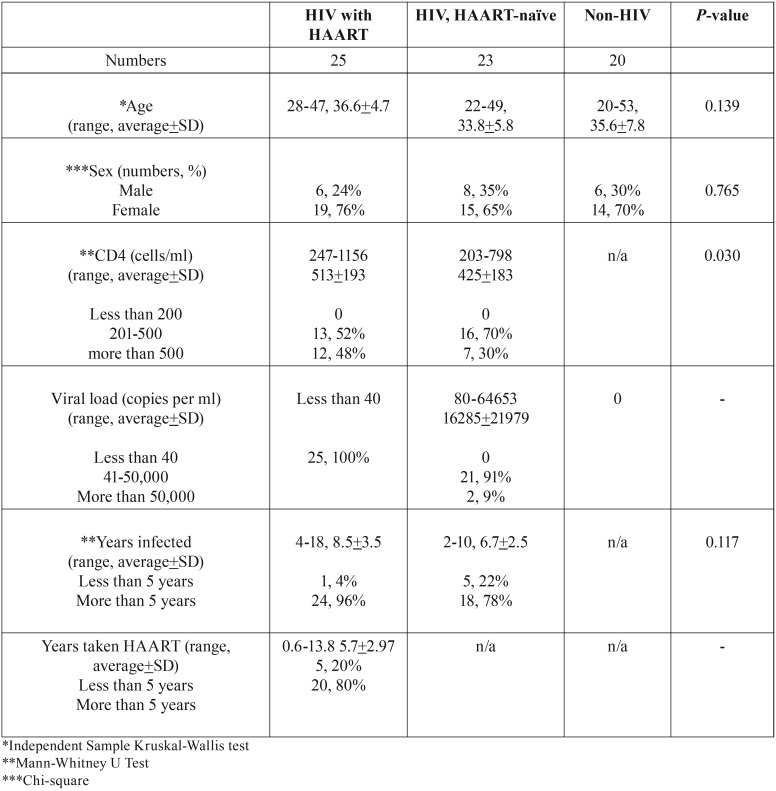
Demographic data of study population.

-Oral and dental findings

Oral lesions observed among HIV-infected subjects included dry mouth and hyperpigmentation. Notably 40% of subjects taking HAART demonstrated hyperpigmentation of oral mucosa, while no hyperpigmentation was found in HAART-naïve subjects. Signs of dry mouth were noted in 20% of HIV-infected subjects taking HAART and 4.3% of HAART-naïve subjects. Other lesions included aphthous ulcer, smoking-induced leukoplakia, nicotinic stomatitis, traumatic ulcer and coated tongue as shown in  Table 2. Noted that these other lesions, except coated tongue, were only observed in HIV-infected subjects taking HAART. However, the numbers were low and may not be of clinical significance.

**Table 2:**
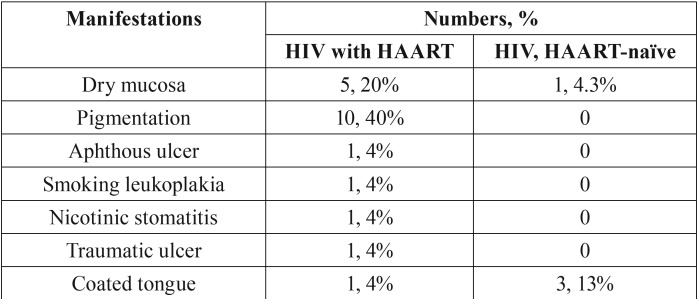
Oral manifestations in HIV-infected subjects.

-Salivary flow rates

Unstimulated salivary flow rates in non-HIV infected subjects (0.60+0.3 ml/min) were higher than HIV-infected groups taking HAART (0.53+0.2 ml/min) and HAART-naïve (0.48+2.2 ml/min). On the other hand, stimulated salivary flow rates in HAART-naïve HIV-infected subjects (3.11+2 ml/min) were higher than non-HIV infected subjects (2.6+1.1 ml/min) and HIV-infected subjects taking HAART (2.34+0.8 ml/min). The summary of data is shown in  Table 3. However, the difference was not statistically significant.

**Table 3:**
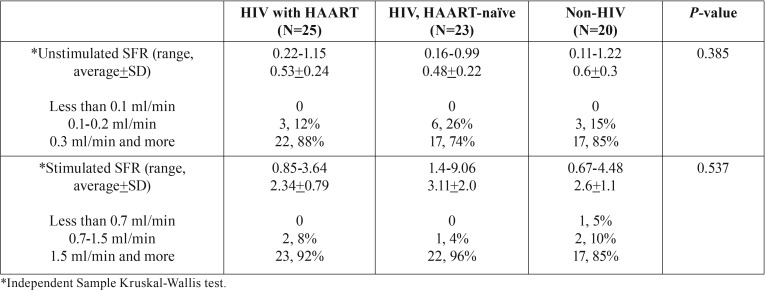
Salivary flow rates (SFR) of study populations.

-*Candida* counts and factors affecting *Candida* colonization

**Figure 1:**
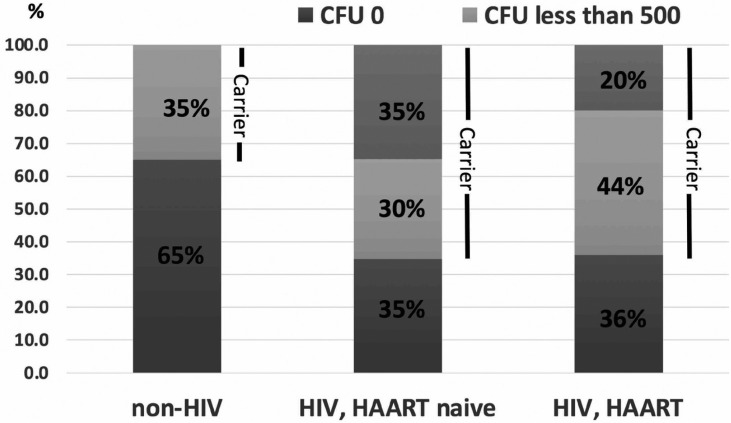
*Candida* carriers and *Candida* counts in non-HIV, HIV-infected subjects who never taken and taking HAART, respectively.

Percentage of *Candida* carriers in HIV-infected subjects taking HAART and HAART-naïve were 64% and 65%, respectively, which were higher than those in non-HIV-infected subjects (35%). In addition, *Candida* CFUs in all non-HIV-infected subjects were lower than 500 CFUs/ml. On the other hand, 20% of HIV-infected subjects taking HAART and 35% of HAART-naïve subjects had CFUs more than 500 CFUs/ml. The summary of data is shown in Figure 1. When we evaluated factors that might affects *Candida* colonization in each group, no statistically difference was found in any factors, including CD4 counts, years of HIV infections, years of HAART usage and salivary flow rates ( Table 4).

**Table 4:**
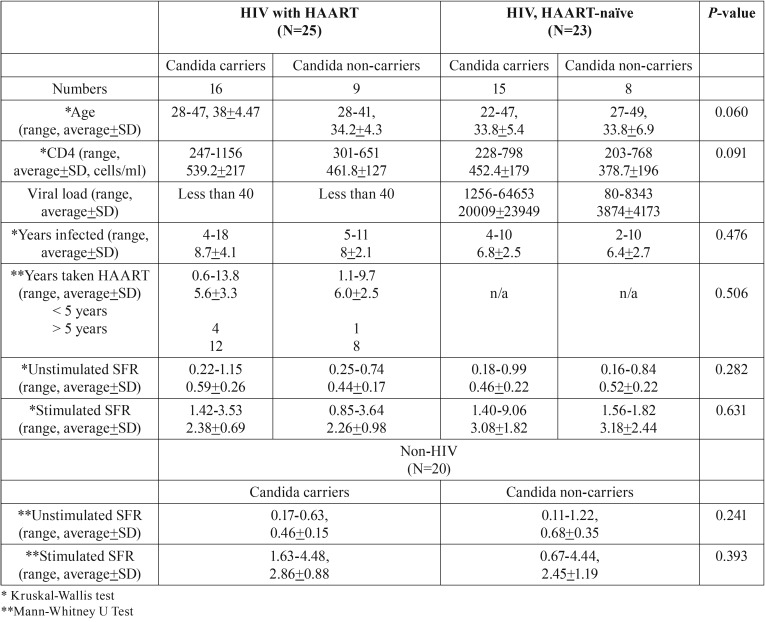
Comparisons of factors between *Candida* carrier and non-carriers.

-*Candida* species

**Figure 2:**
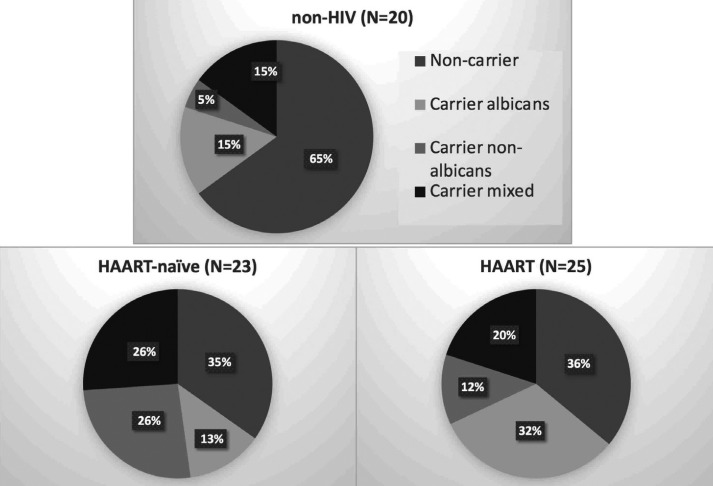
*Candida* species colonization in non-HIV, HIV-infected subjects who never taken and taking HAART, respectively.

Regarding Candida species, HIV-infected subjects with HAART were colonized by *C. albicans* alone (32%) more than mixture between *albicans* and non-*albicans* (20%) and non-*albicans* alone (12%). The profile was similar to non-HIV infected subjects who were colonized mostly by *C. albicans* alone (15%) and mixture between *albicans* and non-*albicans* (15%). On the other hand, majority of *Candida* carriers in HAART-naïve group were colonized by non-*albicans* alone (26%) and mixture between *albicans* and non-*albicans* (26%). Only 13% of them were colonized by *C. albicans* alone. All non-*albicans* species identified in this study demonstrated white colonies, meaning that they were other species than *C. tropicalis* and *C. krusei*. The summary of data is shown in Figure 2. This data suggests that HAART usage and HIV infection might affect *Candida* colonization profile.

## Discussion and conclusion

This cross-sectional study evaluated effects of HIV infection and current HAART regimen on oral conditions and *Candida* colonization in Thai population.

We found that current HAART regimens in majority of our population were combination of NRTIs and NNRTIs, which were different than previously used PIs. This regimen is consistently used nowadays due to reported better virological controls than PIs (6,7). Similar to previous studies, we found that hyperpigmentation is common among HAART users as compared to HAART-naïve and non-HIV-infected subjects. This is not surprising, as hyperpigmentation is a known side effect of HAART and has been reported (1,19). Other oral lesions were few, in accordance to some previous studies (1,19). Another study from Thailand showed higher percentages of oral lesions than our study. However, their subjects had higher viral load levels and lower CD4 counts than our subjects (1). One reason could be because our population received HAART early in their disease courses. This assumption is supported by recent study, which revealed that early administration of HAART when CD4 is lower than 350 cells/ml reduced incidence of oral lesions when compared to late administration (when CD4 is lower than 200 cells/ml) (20). Consistent with previous study in Thailand, we did not find any wart or Human Papillomavirus (HPV)-induced lesion in this population (1). Interestingly, our previous study showed that HIV-infected individuals had higher prevalence of HPVs, especially high-risk HPVs, detected from their oral rinses even without visible lesions (21). Long-term study for prevalence of HPV-associated lesions, including oropharyngeal cancer will be of value.

The prevalence of xerostomia and salivary gland hypofunction has been reported to be 2% to 10% in HIV infected patients (5,22). In addition, previous studies show that PIs led to significant effect on salivary gland function (4,5). On the other hand, continue treatment with non-PI HAART will improve salivary flow rates (5,23). We noted that percentage of HAART users with signs of dry mouth was not different from that of HAART-naïve (24% vs 17.3%). Moreover, salivary flow rates were not statistically different among the three groups. In our study, none of HIV-infected subjects had hyposalivation (less than 0.1 ml/min for unstimulated salivary flow rate and less than 0.7 ml/min for stimulated salivary flow rate). This could be because majority of subjects were long-term users of non-PI-based HAART regimens. Consistent with previous studies, this HAART regimen seems to have little effects to salivary gland function (5,23). However, study by the other group in Thailand showed that salivary flow rates of HAART users were significantly lower than non-HAART users (1). This could be due to differences in saliva collection, HAART regimen and study population. In addition, some of their population had CD4 levels lower than 200 cells/ml, which had been reported to affect salivary gland function in HAART users (1,24).

Our study showed that HIV-infected subjects had higher percentage of *Candida* carriers and *Candida* counts, regardless of HAART status. Percentages of *Candida* carriers in our study were 64% and 65% in HIV-infected subjects taking and not taking HAART, respectively. On the other hand, 35% of *Candida* carriers were detected in healthy control group. In addition, *Candida* colonization CFUs in HIV-infected subjects were higher than those in healthy subjects. The findings were similar to previous studies which demonstrated that *Candida* carriers in HIV-infected subjects ranged from 42-57% (8,9,15). Previous study in Thailand reported 56.6% *Candida* carriers in HIV-infected subjects (9). Previous studies have shown that HAART decreased the incidence of all HIV-related opportunistic infections including fungal infection, but there are conflicting reports regarding the association between oral yeast colonization and HAART. While some studies, including ours, showed no protective effects of HAART on *Candida* colonization, some studies reported decreased in *Candida* colonization in HAART users, particularly those with PI-based regimens (8-10,14,17). These could be due to differences in study population, HAART regimen and fungal identification methods.

While antifungal mechanism of PIs, due to its ability to inhibit fungal secretory aspartyl proteinases (16), is well-known, that of non-PI-based HAART is not clearly understood. Because CD4 levels lower than 200 cells/ml were one of the risk factors of *Candida* colonization, antifungal ability of non-PI-based HAART could be indirectly due to restoration of CD4 levels (15). In our study, although CD4 levels of subjects in HAART-naïve group were significantly lower than those in HAART group, all population had CD4 levels higher than 200 cells/ml. This could explain why we did not see effect of HAART on *Candida* colonization in our population. Table 5 reveled that salivary flow rates and other factors did not affect *Candida* colonization. Other factors might affect -*Candida* colonization in our population.

*Candida* colonization profile observed in our study was similar to other studies. Studies in many countries, including Brazil, Thailand, China, demonstrated that *C. albicans* was the most common species in both healthy and HIV-infected *Candida* carriers. Variety of non-*albicans* species had been detected in HIV-infected *Candida* carriers from 20% to 50% (8,9,25). In addition, mixed colonization had been identified from 7% to 41% (8,9). Percentage of *Candida* species colonization from our findings were within the ranges previously reported. Interestingly, colonization by non-*albicans* and mixed species were observed in healthy subjects. Similar findings were also previously described (8,9).

Risk factors for non-*albicans* colonization are controversial and include reduced CD4 counts (less than 200 cells/ml), use of antibiotics and antifungal drugs, history of tuberculosis, contraceptive use and HIV infection (9,26,27). In our study, HAART-naïve HIV-infected subjects were colonized more by non-*albicans* species. This study showed that although the use of HAART did not reduce *Candida* colonization and *Candida* counts, it led to reduction of non-*albicans* colonization. This was in accordance with previous studies that NRTIs and NNRTIs also had antifungal activity with unknown mechanism (2,7). In addition to antifungal effects of HAART, restoration of CD4 levels might contribute to the reduction of non-*albicans* colonization (26).

Although the role of non-*albicans* and mixed *Candida* colonization in HIV-infected individual is not clear, non-*albicans* species were shown to be more resistant to conventional antifungal drugs (28,29). In addition, mixed infection may make drug resistance more complicated (30). Due to the limited availability of data, certain factors could not be investigated in this study for their impact. Use of contraceptive, particular HAART regimens, history of tuberculosis, nutritional status and drug abuse might affect *Candida* colonization. Future study to identify *Candida* species in larger population and drug sensitivity test will be beneficial.

In conclusion, our study demonstrated that HIV-infected subjects had higher chance to be *Candida* carriers and contained higher numbers of *Candida* colonization than healthy subjects. In addition, HAART usage reduced colonization by non-*albicans* species. Because maintaining low *Candida* counts in this population is important, early HAART administration and appropriate antifungal drugs should be considered in HIV-infected patients.
